# Understanding consumer perception and acceptance of AI art through eye tracking and Bidirectional Encoder Representations from Transformers-based sentiment analysis

**DOI:** 10.16910/jemr.17.5.3

**Published:** 2024-12-22

**Authors:** Tao Yu, Junping Xu, Younghwan Pan

**Affiliations:** Department of Smart Experience Design Kookmin University, Seoul 02707, Republic of Korea

**Keywords:** AI Art, Eye Tracking, Sentiment Analysis, Consumer Perception, Visual Attention, Emotion Analysis, Consumer Acceptance, BERT Model Optimization

## Abstract

This study investigates public perception and acceptance of AI-generated art using an integrated system that merges eye-tracking methodologies with advanced bidirectional encoder representations from transformers (BERT)-based sentiment analysis. Eye-tracking methods systematically document the visual trajectories and fixation spots of consumers viewing AI-generated artworks, elucidating the inherent relationship between visual activity and perception. Thereafter, the BERT-based sentiment analysis algorithm extracts emotional responses and aesthetic assessments from numerous internet reviews, offering a robust instrument for evaluating public approval and aesthetic perception. The findings indicate that consumer perception of AI-generated art is markedly affected by visual attention behavior, whereas sentiment analysis uncovers substantial disparities in aesthetic assessments. This paper introduces enhancements to the BERT model via domain-specific pre-training and hyperparameter optimization utilizing deep Gaussian processes and dynamic Bayesian optimization, resulting in substantial increases in classification accuracy and resilience. This study thoroughly examines the underlying mechanisms of public perception and assessment of AI-generated art, assesses the potential of these techniques for practical application in art creation and evaluation, and offers a novel perspective and scientific foundation for future research and application of AI art.

## Introduction

In recent years, rapid advancements in AI and its growing applications in art have attracted considerable attention from academia and industry alike, driving innovation and blending traditional creative forms (1; 2; 3). AI algorithms empower artists to explore novel production techniques and creative expressions, surpassing traditional artistic boundaries (44). The creation and reception of AI-generated art are shaped by advancements in technology and shifts in consumer attitudes (40). As AI technologies redefine artistic creation, understanding the role of visual behavior and emotional responses in shaping public perception and acceptance becomes crucial (10). This study tackles these issues by combining eye-tracking methodologies and sentiment analysis, providing fresh perspectives on the nexus of technology, art, and consumer psychology (21; 24; 42).

AI art is a nascent domain that amalgamates artificial intelligence technology with artistic expression. It focuses on the application of AI algorithms and methodologies to autonomously or collaboratively produce artworks. AI art encompasses the re-creation of conventional art forms, including visual art, music, and literature, as well as transnational art forms like new media art and interactive art. From a technical perspective, AI art employs sophisticated algorithms, including machine learning, deep learning, and generative adversarial networks (GANs), to create artistically valuable pieces by studying and learning from extensive datasets. These works may be produced solely by AI or through collaboration between human artists and AI (37). The utilization of AI in artistic creation has advanced considerably, including several domains like visual arts, music composition, literary production, and interactive art. The current research findings primarily concentrate on the following elements. The utilization of deep learning methodologies (23), particularly Generative Adversarial Networks (GANs), in artistic production has garnered significant interest and thorough investigation (17). Generative Adversarial Networks (GANs) have attained significant success in picture generation, style transfer, and video production, serving as a potent instrument for the creation of visual art. Furthermore, AI has achieved considerable advancements in music composition, with several research investigating the use of deep learning for melody production, harmonic structuring, and music style emulation (41). OpenAI's MuseNet and Google's Magenta initiatives have showcased AI's capability to compose in many musical styles, facilitating the sophisticated evolution of music composition (34). Research in literary creation emphasizes the utilization of Natural Language Processing (NLP) technology, with extensive language models like GPT-3 exhibiting significant potential in text production, poem composition, and content development (28). The synthesis of the aforementioned studies indicates that the evaluation criteria for AI artworks remain ambiguous, predominantly depending on subjective judgment and lacking a systematic and objective assessment framework (45). Establishing a scientific assessment method to measure and evaluate the quality and artistic worth of AI-generated artworks is an important issue that requires resolution.

Eye tracking is a method employed to document and examine the trajectories of human eye movements (18). This approach elucidates the allocation of an individual's visual attention and cognitive processes within a certain activity or environment by tracking the position and movement of the eye on a visual stimulus. Eye-tracking methodologies have been extensively employed in behavioral research across several domains, including psychology, cognitive science, human-computer interface, and marketing (20). Eye tracking is employed in psychology and cognitive science to examine visual attention, information processing, and cognitive load. For instance, by examining gaze positions and scanning trajectories, researchers might elucidate the cognitive processes and information synthesis techniques employed while reading (14). In human-computer interaction research, eye tracking facilitates the design and assessment of user interfaces to enhance layout and interaction flow by analyzing users' visual behavior (43). In marketing, eye tracking is employed to examine the allocation of customer attention and the decision-making process, assisting organizations in refining advertising design and product presentation methods (5). For instance, literature employed eye-tracking technology to investigate the visual perception of educators and learners during instructional activities to improve student engagement and focus in the classroom (35). The study examined the influence of online product evaluations on customer purchasing behavior through the use of eye tracking equipment (9). The areas of customers' gaze throughout the purchasing decision process were examined to identify the zones of interest. The utilization of eye-tracking technology in art perception research offers an innovative method for comprehending how viewers see and assess artworks. Recording and evaluating audience eye movement trajectories while observing artworks reveals the distribution pattern of visual attention and the perceptual process, resulting in a greater comprehension of the artworks' appeal and emotional impact (8).

Sentiment analysis is a significant domain within NLP, primarily utilizing algorithms and technological methods to autonomously identify and extract sentiment information from text (38). Sentiment analysis include the classification of sentiments as positive, negative, or neutral, along with the evaluation of sentiment polarity and intensity (12). Sentiment analysis offers novel study methodologies and instruments for the disciplines of social sciences, humanities, and computer science. Researchers can utilize sentiment analysis to explore the transmission processes and determinants of social emotions, comprehend the emotional reactions of various groups throughout certain occurrences, and uncover social psychological and behavioral patterns. Conventional sentiment analysis techniques primarily encompass dictionary-based approaches (15) and machine learning-based methodologies (31). Lexicon-based approaches fundamentally involve analyzing text using a pre-established sentiment lexicon. Literature (36) presents a text sentiment analysis methodology that amalgamates several variables through the development of dictionary-based sentiment and expression attributes, subsequently creating an associated text sentiment classification model. Literature (19) contends that dictionary-based sentiment analysis methods have significant shortcomings when compared to several other approaches. Sentiment lexicons exhibit restricted coverage and provide challenges in adapting to the diversity and evolving nature of language. This strategy is less reliable and susceptible to misjudgment when addressing context-dependent and intricate sentiment expressions (27). Machine learning methodologies have been established within the framework of big data and enhanced computing capabilities, which autonomously detect and classify emotion information in text through model training. Literature (4) examined possible alterations in neuronal communication networks during emotional fluctuations using brainwave analysis utilizing several machine learning methodologies. Machine learning methodologies for sentiment analysis can manage extensive text datasets and exhibit significant adaptability (33). Nonetheless, some limitations are inherent to machine learning-based methodologies. The model's training method necessitates a substantial quantity of labeled data, and its interpretability is limited, complicating the intuitive comprehension of its classification outcomes (22). Moreover, learned models frequently require retraining or adaptation when encountering new domains or languages, hence augmenting the complexity of actual implementations (25).

As artificial intelligence technology advances rapidly, sentiment analysis techniques have progressively transitioned from classic lexicon and machine learning approaches to more sophisticated deep learning methodologies. Deep learning, by mimicking the architecture of human brain neural networks, can autonomously extract intricate information from text, hence enhancing the precision and resilience of sentiment analysis (30). The BERT model has emerged as a focal point of study owing to its robust language comprehension capabilities and extensive applicability. The BERT model has emerged as a prominent study subject because to its robust language comprehension abilities and many potential applications (11). BERT intricately encodes contextual information via the bi-directional Transformer architecture to provide more precise word vector representations (16). In sentiment analysis, the BERT model undergoes initial pre-training on extensive text data, followed by fine-tuning on targeted sentiment analysis datasets, therefore enhancing classification accuracy dramatically. The BERT model is extensively utilized in social media comment analysis, user feedback sentiment identification, and market research (32). By assessing the sentiment of social media comments, we may promptly understand the people's emotional attitudes and mood fluctuations on significant events, therefore aiding in public opinion monitoring and crisis management. The BERT model effectively discerns consumers' emotional assessments of items and services in sentiment recognition, aiding firms in enhancing product design and service quality (7). Literature (1) presents a technique utilizing natural language processing to classify news stories. The technique is executed using transfer learning utilizing the BERT language model of Transformers.

Motivated by the above research, the essay provides a novel approach to examine public perception and acceptance of AI-generated art through the utilization of eye-tracking technology and BERT-based sentiment analysis. The primary innovations of the paper may be encapsulated as follows: The paper establishes a comprehensive framework by integrating psychology, computer science, and linguistics, combining eye-tracking technology with the BERT model to analyze visual attention and emotional responses simultaneously. Eye-tracking captures participants' visual patterns, such as gaze points, saccades, and dwell duration, revealing the connection between visual attention and emotional reactions (13). In sentiment analysis, the study enhances classification performance by developing Sentiment BERT and Emotion BERT models, optimized through domain-specific pre-training and dynamic Bayesian optimization, improving precision and adaptability in varying contexts.

This study seeks to explore two primary research questions: (1) In what ways do consumers' visual attention patterns influence their perception of AI-generated art? (2) How do emotional reactions, identified through sentiment analysis, affect the acceptance of such art by consumers? To investigate these questions, we employ an innovative framework integrating high-precision eye-tracking with a BERT-based sentiment analysis model. The objective is to gain a holistic understanding of how visual behavior and emotional responses shape public reception of AI art, while also establishing a solid methodological foundation for future research in this field.

## Methods

The essay methodically examines customer perception and acceptance of AI-generated artwork with eye-tracking technology and BERT-based sentiment analysis techniques.

**Figure 1.**
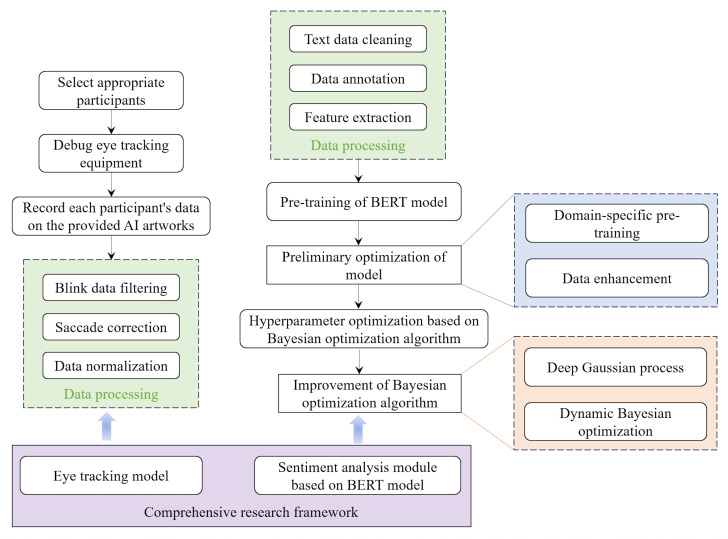
Integrated research framework

The study investigates consumer perception and acceptance of AI-generated artwork by developing a robust research framework that integrates theories and methodologies from psychology, computer science, and linguistics (26), facilitating the analysis of visual attention and emotional responses via eye-tracking technology and BERT modeling (39) . The article presents an integrated framework utilizing eye-tracking and BERT modeling, illustrated in Figure 1.

The suggested technique encompasses the subsequent essential components: Eye-tracking data collection involved recording participants' visual behavioral data as they observed AI-generated artwork using high-precision eye-tracking sensors. Collection of emotional reaction data: gathering textual data from participants' online comments post-viewing for sentiment analysis. Data preprocessing: the cleaning and preparation of gathered eye movement and text data to guarantee accuracy and uniformity. Data analysis: Examination of visual behavior and emotional responses using preprocessed data to elucidate their influence on the perception and acceptance of artistic excellence.

### Eye Tracking Module

This article use eye-tracking technology to record and examine participants' visual activity while viewing AI-generated artwork. The advanced eye-tracking apparatus and data collecting and analysis techniques effectively elucidated the visual attention patterns and their influence on the perception of artistic quality.

### Equipment used

The Tobii Pro Spectrum eye tracker was employed to acquire eye movement data with exceptional accuracy. The apparatus has a fast sampling rate, exceptional precision, and a configuration devoid of interference. The Tobii Pro Spectrum, with a sampling rate of up to 1200 Hz, captures eye movement data with exceptional spatial and temporal precision, correctly documenting a participant's gaze point and trajectory. The gadget is simple to configure and facilitates data collecting in an interference-free setting, guaranteeing data precision and dependability.

### Experimental ideas

To guarantee the accuracy of the data collected for the article, the eye-tracking experiment was executed in a tranquil, distraction-free laboratory setting. Participants were instructed to position themselves at a predetermined distance from a display to guarantee the precise recording of their eye movements by the eye-tracking gadget. Participants observed the AI-generated artwork, which was exhibited for 30 seconds. One hundred participants, aged 18 to 35, were recruited using an online platform to guarantee a varied sample. Participants were shown a total of 10 AI-generated artworks. This ensured a consistent viewing experience and allowed sufficient time for visual exploration and fixation identification. A fixation was defined as a period during which the gaze remains on a specific point for at least 300 milliseconds, which aligns with common standards in eye-tracking research.

### Data processing

Eye movement data acquired experimentally must undergo thorough cleaning and preprocessing to guarantee data quality and dependability. The stages of data processing encompass the following:

Data filtering during blinks: data collected during blinks typically lacks analytical significance and must be excluded in the data processing phase. By identifying blink occurrences and excluding the associated time intervals, this segment of the extraneous data may be eliminated. Specifically, the detection of blinking events can be realized by setting the velocity threshold $\theta_{v}$, when the eye movement velocity v(t) exceeds $\theta_{v}$ , it is regarded as a blink and the data of that time period is excluded.

Sweeping correction: Sweeping is a process of rapid eye movement, during which the data points on which the line-of-sight rests may be inaccurate. The accuracy of the data can be improved by identifying and correcting the data during the sweep. In the analysis, saccades (previously referred to as “sweeps”) were identified to capture rapid eye movements between fixation points. Saccades provide insights into how participants shift their visual attention across different regions of the artworks.

Data normalization: To ensure consistency across participants using different screen sizes, we applied data normalization. Additionally, this paper represented key results in visual degrees, a unit commonly used in eye-tracking studies, to enhance comparability and interpretation. Since there may be differences in the screen size and resolution used by different experimental participants, data need to be normalized to ensure comparability and consistency. The effects of these differences can be eliminated by converting all data points (x,y) into a uniform coordinate system $\left(x^{\prime },y^{\prime }\right)$. The normalization formula is as follows:

**(1):**
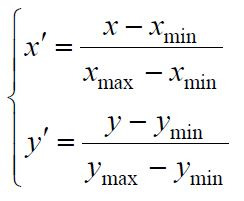


where $x_{\min}$ and $x_{\max}$ denote the minimum and maximum values of *x*; $y_{\min}$ and $y_{\max}$ denote the minimum and maximum values of *y*, respectively.

### BERT-based Sentiment Analysis Approach

This paper fine-tuned the BERT model using domain-specific datasets, focusing on improving sentiment classification accuracy. While technical optimizations such as hyperparameter tuning were applied, the emphasis of our study lies in integrating sentiment analysis with eye-tracking insights to explore consumer perception.

### Training and Parameter Optimization of the BERT Model

The pre-trained BERT model was developed using an extensive text corpora (Books corpora), acquiring a comprehensive language representation using the Masked Language Model (MLM) and Next Sentence Prediction (NSP) tasks. Each input sequence comprises three components: word embedding, positional embedding, and segmental embedding, which collectively capture lexical and syntactic information.

We refined the pre-trained BERT model for sentiment analysis of articles by domain-specific pre-training and optimization. A sentiment classification dataset was compiled and annotated to categorize textual comments into positive, negative, and neutral categories. All textual data was converted into the BERT input format, incorporating special tokens ([CLS] and [SEP]) and mapping each word to word embeddings. An Adam optimizer is employed during training with an initial learning rate of 2e-5, and a cross-entropy loss function is utilized to reduce classification errors. The training is conducted on a GPU with a batch size of 32 and four epochs. The cross-entropy loss function is defined as follows:

**(2):**
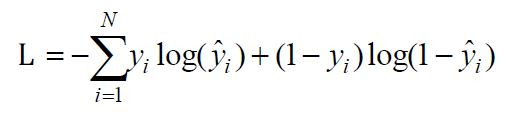


where L is the cross-entropy loss, $y_{i}$ is the actual label, ${\hat{y}}_{i}$ is the predicted probability, and N is the total number of samples.

The study employs a domain-specific pre-training and data-enhanced optimization technique to enhance the performance of the BERT model in sentiment classification tasks. In the initial stage, supplementary pre-training is conducted on a domain-specific corpus pertinent to sentiment analysis to assimilate domain-specific linguistic patterns. This method can enhance the model's comprehension and processing of sentiment-related material, hence improving the precision of sentiment categorization. Secondly, data augmentation techniques are employed to augment the diversity of training data. Data augmentation methodologies encompass two primary techniques: back-translation and synonym substitution. Back-Translation is a technique that produces new text with varied phrases but retaining the same meaning by translating the original text into a different language and subsequently translating it back to the original language. This approach can enhance the diversity of training data and augment the model's generalization capability. Synonym Replacement produces new training data by substituting terms in the text with their synonyms. This approach can enhance data variety while preserving the original meaning, thereby augmenting the model's generalization capability.

### Data processing

Prior to doing sentiment analysis, the gathered data must undergo preprocessing to guarantee its quality and consistency. Data preparation include procedures such as text data cleansing, annotation, and feature extraction:

Text data cleaning: involves the elimination of stop words, punctuation, HTML elements, and infrequent terms. Discontinued words are terms that regularly occur in the text but lack practical relevance for sentiment analysis, such as "the," "is," and similar words. Eliminating obsolete terminology helps diminish extraneous noise and enhance the model's precision. Eliminating deactivated words can decrease noise and enhance the model's accuracy.

Data labeling: the purified text data are assigned sentiment labels and categorized into three classifications: positive, negative, and neutral. Data labeling serves as the foundation of sentiment analysis, guaranteeing that each text datum possesses a distinct sentiment label.

Feature extraction: each text datum is transformed into the input format suitable for the BERT model. The precise procedure involves including specialized tags and associating each word with a BERT word embedding. The word embeddings are produced using a pre-trained BERT model that encapsulates the contextual information of the words.

Data Augmentation: Employing back-translation and synonym substitution methods to enhance the variability of training data. Back-translation involves translating an original text into a foreign language and subsequently translating it back to the original language, resulting in a new text that conveys the same content but employs different phrases. Synonym substitution involves replacing terms in the text with their synonyms to provide new training data. These strategies can enhance data variety and augment the model's generalization capability.

### Optimization of the hyperparameters of the BERT model

To enhance the performance of the BERT model in sentiment classification, the study employs a Bayesian optimization approach to adjust the model's hyperparameters.

### Steps in Bayesian optimization algorithm

Bayesian optimization is a global optimization technique that approximates an objective function by developing an agent model and identifying the best hyperparameter combination based on this model. Bayesian optimization estimates the objective function by developing an agent model, often a Gaussian process, and using this model to determine the subsequent sample location, so identifying the best value of the objective function with minimal evaluations. The optimization procedure is outlined as follows: Initially, an agent model is developed to estimate the goal function, utilizing the assessed hyperparameter combinations and their associated objective function values. Subsequently, utilizing the agent model and the acquisition function (e.g., Expected Improvement function, EI), the subsequent hyperparameter combination for evaluation is determined. The newly selected hyperparameter combination is subsequently assessed, and the agent model is revised with the updated data points. The aforementioned stages are reiterated till the termination criterion is satisfied.

The efficacy of the BERT model in sentiment classification tasks is significantly influenced by the choice of hyperparameters. The Bayesian optimization approach systematically explores the hyperparameter space to identify optimal combinations that enhance model performance. The procedure to enhance the hyperparameters of the BERT model utilizing the Bayesian optimization technique is outlined as follows:

Define the hyperparameter space: identify the hyperparameters for tuning and their respective value ranges. Common hyperparameters for the BERT model encompass learning rate, batch size, and the number of training epochs:

**(3):**
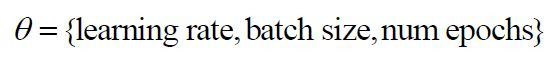


Construct the objective function: the objective function is the loss or accuracy of the model on the validation set. Denoted as f(θ), it is of the form:

**(4):**
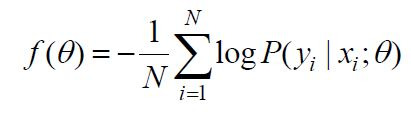


where N is the number of samples in the validation set and $NP\left(y_{i}|x_{i};\theta \right)$ is the probability that the model predicts the i -th sample under the parameter θ.

Initialize the agent model: construct the agent model based on Gaussian process, denoted as $\hat{f}\left(\theta \right)$ , which is of the form:

**(5):**
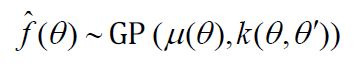


where μ(θ) is the mean function and $k\left(\theta ,\theta^{\prime }\right)$ is the covariance function.

Selection of sampling location: select the next combination of hyperparameters to be evaluated based on the desired improvement function $\theta_{\text{next}}$ , which is of the form:

**(6):**
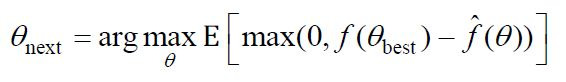


where $\theta_{\text{best}}$ is the current optimal hyperparameter combination.

Evaluate and update the agent model: evaluate the objective function value $f\left(\theta_{\text{next}}\right)$ corresponding to $\theta_{\text{next}}$ and update the agent model.

Iterative optimization: repeat steps 4 and 5 until the termination condition (maximum number of iterations or convergence of the objective function) is met.

Through the above process, the Bayesian optimization algorithm can efficiently search the hyperparameter space and find the optimal hyperparameter combinations that enhance the performance of the BERT model. The method not only improves the accuracy of the model, but also significantly reduces the time and computational resources for hyperparameter tuning.

### Improvement of Bayesian Optimization Algorithm

To enhance the hyperparameter tuning efficacy of the BERT model in sentiment classification tasks, the article proposes an enhancement to the Bayesian optimization algorithm, incorporating concepts from deep Gaussian processes and dynamic Bayesian optimization to improve the algorithm's optimization performance.

Deep Gaussian process, conventional Gaussian processes are ineffective in managing high-dimensional data and struggle to accurately represent intricate high-dimensional data distributions. Deep Gaussian processes more effectively describe intricate nonlinear interactions with the use of a multilayer architecture. Every layer of a deep Gaussian process constitutes a Gaussian process, with its output functioning as the input for the subsequent layer. The output of a deep Gaussian process for a certain input may be articulated as:

**(7):**
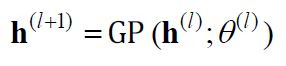


where ${𝐡}^{\left(l\right)}$ represents the output of layer l and $\theta^{\left(l\right)}$ is the parameter of layer l.

Dynamic Bayesian optimization, during the optimization process, the objective function may change over time. Dynamic Bayesian optimization dynamically adjusts the agent model and sampling strategy to adapt to the changes in the objective function by introducing the time dimension. Specifically, the objective function can be expressed as f(𝐱,t) , where t is time. The objective of dynamic Bayesian optimization is to find the optimal combination of hyperparameters in each time period.

Consequently, the integration of deep Gaussian processes with dynamic Bayesian optimization yields crucial steps outlined in the research to augment the efficacy of the BERT model in sentiment classification tasks using an enhanced Bayesian optimization approach:

Construct a deep Gaussian process model: Based on the evaluated hyperparameter combinations and their corresponding objective function values, a multilayer Gaussian process model $\hat{f}\left(𝐱;\theta \right)$ is constructed to approximate the objective function.

**(8):**
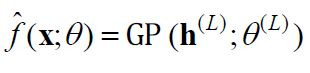


where ${𝐡}^{\left(L\right)}$ is the output of the last layer and $\theta^{\left(L\right)}$ is the parameters of the last layer.

Introducing time dimension: time t is introduced as an additional input dimension to the deep Gaussian process model to construct the dynamic agent model $\hat{f}\left(𝐱,t;\theta \right)$:

**(9):**
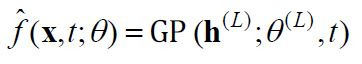


Selecting the sampling location: based on the dynamic agent model and the expected improvement function, select the next combination of hyperparameters to be evaluated $\left({𝐱}_{\text{next}},t_{\text{next}}\right)$ :

**(10):**
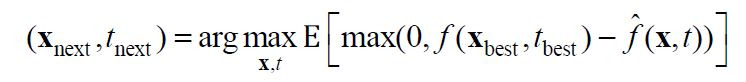


where ${𝐱}_{\text{best}}$ and $t_{\text{best}}$ are the current optimal hyperparameter combinations and time points.

Evaluate and update the agent model: evaluate the objective function values $f\left({𝐱}_{\text{next}},t_{\text{next}}\right)$ corresponding to the new hyperparameter combinations $\left({𝐱}_{\text{next}},t_{\text{next}}\right)$ and update the deep Gaussian process model with the new data points.

Iterative optimization: repeat steps 3 and 4 until the termination conditions (e.g., maximum number of iterations or convergence of the objective function) are met.

By integrating deep Gaussian processes with dynamic Bayesian optimization, the study successfully optimizes the hyperparameters of the BERT model in fluctuating settings, hence enhancing the performance of the sentiment classification task. This suggested new technique boosts both the efficiency and accuracy of the optimization process, while also improving the model's flexibility in real applications.

## Experimental design

To get more precise test outcomes and mitigate the detrimental impacts of random variables, the subsequent experimental framework is devised to enhance the accuracy and reliability of the results.

### Sample selection method

To guarantee the representativeness and external validity of the model presented in the paper, the sample selection technique was meticulously crafted regarding sample characteristics and size during the selection of the test sample.

### Sample characteristics

The objective of the article's research is to investigate consumer perception and acceptability of AI-generated artwork; hence, the sample must be sufficiently broad and representative to encompass customers with varying demographic features. The following traits are enumerated below:

A. Age: Participants were aged between 18 and 35 years. This age range was selected due to this demographic's generally elevated acceptance and enthusiasm for developing technology and digital arts.

B. Gender: The sample was evenly distributed between males and females to guarantee that the study's outcomes were not influenced by gender disparities.

C. Educational level: Participants possess varied educational qualifications, ranging from high school and lower to college and graduate degrees and beyond. Individuals with varying educational backgrounds may possess differing degrees of comprehension and acceptance of AI-generated art.

D. Cultural context: Participants from diverse cultural backgrounds were recruited to examine the influence of cultural disparities on the perception and acceptance of AI-generated artwork.

The sample consisted of 100 participants, with a balanced gender distribution (50 males and 50 females). Participants were aged between 18 and 35 years, a demographic known for higher familiarity with digital technologies and greater acceptance of AI-generated content. The sample was selected through an online recruitment platform, and participants were randomly assigned to different presentation orders to minimize order effects.

### Sample size

The sample size significantly influences the study's reliability. To guarantee the consistency and reliability of the statistical outcomes, 100 participants were enlisted for this investigation. The sample size was established according to the following considerations:

A. Statistical significance: Ensure that the sample size is large enough to obtain statistically significant results through the sample size formula. Assuming that the effect size to be tested in the study is δ , set the significance level α and test efficacy β , the sample size formula is as follows: 

**(11):**
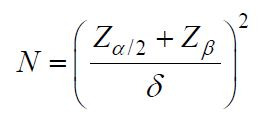


where $Z_{\alpha /2}$ is the quantile corresponding to the significance level α/2 under the standard normal distribution, $Z_{\beta }$ is the quantile corresponding to an efficacy of β , and δ is the effect size. Using this formula, the article determined a sample size of 100 participants.

B. Sample diversity: Ensure that the sample is sufficiently diverse to reflect different demographic characteristics and backgrounds. Volunteers were recruited through an online platform and a screening questionnaire was used to confirm that they met the requirements of the study.

### Data collection and management

To guarantee the precision and comprehensiveness of the data, the article implements the collection and management of ocular movement data and emotional data using the following methodologies.

### Data Acquisition

All participants viewed the artworks on a 24-inch monitor with a resolution of 1920x1080 pixels, placed at a fixed distance of 60 cm from the participants to ensure consistency in viewing conditions. Due to potential differences in individual screen calibrations and participants' seating positions, we applied data normalization to ensure comparability. Normalization adjusted the raw eye-tracking coordinates into a unified scale, enhancing the precision of gaze pattern analysis.

Eye movement data were acquired in a distraction-free laboratory setting with a Tobii Pro Spectrum eye tracker with a sampling rate of 1200 Hz. Participants were positioned at a predetermined distance in front of a display, with the eye-tracker affixed beneath the display to guarantee precise acquisition of eye movement data. Participants sequentially observed a collection of AI-generated artworks, each exhibited for 30 seconds, during which the eye-tracker documented the participant's gaze point, sweep route, and dwell duration. Prior to the official experiment, the eye-tracker was calibrated to guarantee the precision and dependability of the data. The paper gathered high-precision eye movement data through these procedures, establishing a foundation for later visual behavior research.

### Collection of emotional data

Emotional data were acquired by gathering textual responses from participants' online comments following their viewing of the artworks. Participants completed an online questionnaire immediately after viewing each artwork, detailing their emotional responses to it. The survey comprised open-ended inquiries and an emotional assessment scale. Participants' remarks were gathered and preserved for future sentiment analysis. The questionnaire design guaranteed that the variety and depth of the responses yielded adequate data for sentiment analysis.

### Data Administration

Data management encompasses data storage and protective measures to guarantee data security and privacy. All gathered data is stored in encrypted databases to guarantee data integrity and security. Storage protocols encompass routine data backups to avert data loss. Backup data is retained on an external server to guarantee data security. Access to the database is limited to authorized workers to avert unauthorized access and data breaches. All data access is documented for auditing reasons. Furthermore, many tiers of data security were implemented to safeguard the privacy of individuals. Participants' personal information was anonymised prior to data storage to guarantee that they could not be recognized through the data. SSL/TLS encryption was employed during data transfer to avert theft or tampering of data in transit. A privacy statement was presented to participants before to their involvement in the experiment, including the intended use of the data and the protective measures used to guarantee informed consent.

### Definition of variables and setting of indicators

The paper delineates several dependent and independent factors to thoroughly examine customer perception and acceptance of AI-generated artwork.

### Dependent Variables

Perception quantifies the participant's subjective assessment of the overall quality and artistic merit of the AI-generated artwork. It conveys the participants' profound sentiments on the artwork on both visual and emotional dimensions. The article measures participants' impressions with a questionnaire that examines their assessments of the artwork's visual appeal, inventiveness, innovation, production procedures, and complexity. Each aspect was rated on a scale ranging from 1 to 7, with 1 indicating very dissatisfied and 7 indicating very satisfied. The final perceptual ratings Q were calculated by a weighted average method:

**(12):**
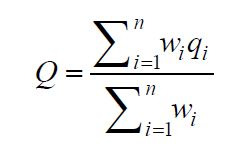


where $q_{i}$ denotes the rating of the i -th aspect, $w_{i}$ denotes the corresponding weight, and n is the number of aspects evaluated.

Acceptance quantifies participants' approval of AI-generated artwork, encompassing their readiness to purchase, endorse, and revisit it. Acceptance signifies participants' perceptions of the artwork about practical implementation and market viability. The paper measures participants' approval using a questionnaire comprising three components: their willingness to purchase the artwork, their willingness to promote the artwork to others, and their willingness to examine the artwork again. Each dimension was evaluated on a scale from 1 to 7, where 1 represented not at all willing and 7 denoted highly willing. The final acceptance score was determined using a weighted average technique:

**(13):**
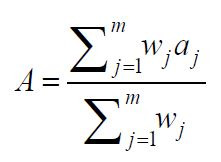


where $a_{j}$ denotes the rating of the j th aspect, $w_{j}$ denotes the corresponding weight, and m is the number of aspects evaluated.

### Independent variables

To thoroughly investigate the impact of visual behavior and emotional reaction on customer perception and acceptability, the paper delineates two categories of independent variables: visual behavioral indicators and emotional analysis indicators.

(1) Visual Behavioral Indicators

Visual behavioral measurements were acquired using eye-tracking technology and encompass the following:

Gaze Duration: The time participants allocated to each gaze point was measured to evaluate their attentiveness to various regions. Increased gaze length correlates with heightened attention to the region. The length of gazing may be articulated as:

**(14):**
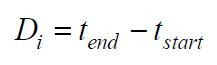


where $t_{start}$ and $t_{end}$ are the start and end times of the gaze, respectively.

Sweep length: Participants' sweep distances between different gaze points were measured to analyze the trajectory of their visual attention. Longer sweep distances may indicate that participants frequently switched attention between different regions. Sweep length can be expressed as:

**(15):**
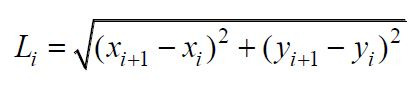


where $\left(x_{i},y_{i}\right)$ and $\left(x_{i+1},y_{i+1}\right)$ are the coordinates of neighboring gaze points *i* and i+1, respectively.

Areas of Attention: By plotting the data on gaze points into a heat map, it is possible to visualize the areas of attention that participants focused on when observing the AI-generated artwork. The brighter the color in the heatmap, the more attention the area received. The article uses the following 2D Gaussian function to smooth the gaze points to generate the heat map:

**(16):**
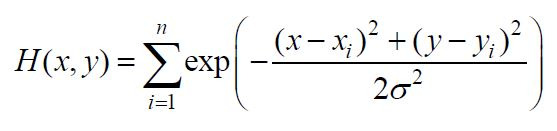


where σ is the smoothing parameter.

(2) Sentiment analysis indicators

Sentiment analysis metrics are obtained through the BERT-based sentiment analysis model, which consists of the following aspects:

Sentiment category: indicates the overall emotional response of participants to the AI-generated artwork, which is categorized into three categories: positive, negative, and neutral. The affective categories were categorized by the Sentiment BERT model with the following prediction formula:

**(17):**
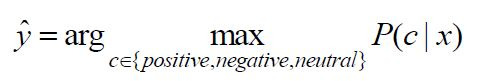


where $\hat{y}$ is the predicted sentiment category and P(c|x) denotes the probability of the sentiment category c given the text x .

Emotional intensity: indicates the intensity of the participants' emotional response to the AI-generated artwork. Emotion intensity was analyzed at a fine-grained level through the Emotion BERT model to quantify the intensity of each emotion category. The goal of the emotional intensity evaluation was to determine the emotional intensity score for each text:

**(18):**
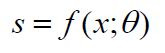


where s is the sentiment intensity score, x is the input text, θ is the model parameters, and f is the model function.

Sentiment Distribution: Statistical analysis of the classification results to calculate the distribution of each sentiment category and the distribution of sentiment intensity. Sentiment distribution statistics can reveal the distribution of emotional tendency and intensity in text data:

**(19):**
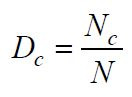


where $D_{c}$ is the distribution of the sentiment category c, $N_{c}$ is the number of texts in the sentiment category c, and N is the total number of texts.

By defining and measuring these independent variables, it is possible to systematically analyze the impact of visual behavior and emotional response on consumer perception and acceptance, providing a scientific basis for subsequent data analysis and research conclusions.

## Analysis of results

This paper comprehensively analyzes the test results from the following two aspects: eye tracking and emotion analysis based on BERT model.

### Analysis of eye tracking results

In eye-tracking data analysis, the examination of visual attention patterns is a crucial method for comprehending participants' visual activity when viewing AI-generated artwork. The paper examines the aforementioned behaviors using visual path analysis and gaze point analysis.

### Analysis of visual attention patterns

Visual path analysis revealed the way participants' visual attention shifted between regions of the artwork by tracking their visual movement trajectories. Based on the preprocessed eye movement data, the visual path of each participant was determined. The sight path consisted of a series of chronologically ordered gaze points $\left(x_{i},y_{i}\right)$. Afterwards, the participants' sight paths were plotted to form a gaze path map.

**Figure 2.**
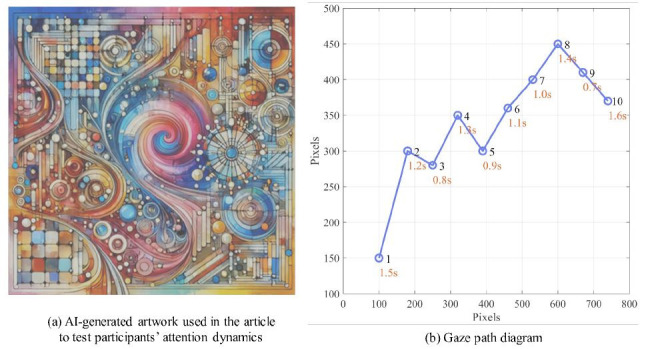
Gaze path diagram of a typical participant

Using the AI-generated artwork seen in Fig. 2(a) as the subject (with the lower left corner of the image as the coordinate origin), Fig. 2(b) illustrates the gaze trajectory of a participant during the observation procedure. Figure 2(b) indicates that the participant's gaze trajectory exhibits distinct stage features. The gaze locations were predominantly focused in the central and higher areas of the artwork, signifying a significant visual appeal for the individual. The distance and direction of visual movement between each pair of gaze locations differed, with a reduced distance from gaze point 4 to gaze point 5, suggesting that individuals exhibited heightened observation in this area. The extended distance traversed from gaze point 7 to gaze point 8 indicated the participants' swift scanning procedure over the various locations. The distribution of gaze duration elucidates the duration of participants' gaze across various locations. Gaze locations 1, 8, and 10 had prolonged gaze durations of 1.5 s, 1.4 s, and 1.6 s, respectively. These areas may have more intricate or prominent visual components, resulting in prolonged observation and processing by participants. Gaze point 3 and gaze point 9 had reduced gaze durations of 0.8 s and 0.7 s, respectively, indicating that these areas may have been less visually engaging to the participants, who conducted only a cursory examination. The analysis of gaze paths and gaze durations revealed significant disparities in the visual appeal of various regions of the AI-generated artwork to the participants, offering crucial data for comprehending consumer perceptions of such artwork. In Figure 2(b), we present a sample visual trajectory with 10 fixation points for clarity. These points represent a typical participant’s viewing path, chosen to illustrate the detailed movement patterns observed. The complete trajectory data for all participants is provided in Appendix A to ensure transparency and reproducibility.

**Figure 3.**
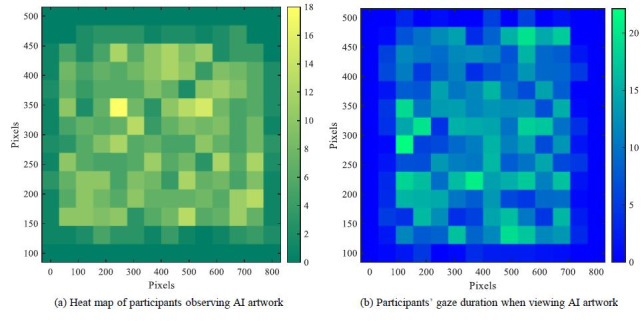
Test results of 100 participants' gaze point and gaze duration

Point-of-attention analysis examines the duration participants remain in a certain region and the allocation of their attention points. Analyzing point-of-attention data enables the identification of regions that captivate the most interest from participants. Point-of-attention analysis primarily involves data about the allocation of attention points relative to gaze duration distribution. The article examined the visual attention data of 100 participants while scrutinizing the AI-generated artwork in depth, and by counting the coordinates of each gaze point $\left(x_{i},y_{i}\right)$ data, the heat map of visual attention and the distribution of gaze time shown in Figure 3 were drawn. The brighter color in the graph indicates the more concentrated attention points, i.e., the region is more visually attractive to the participants. Figure 3(a) indicates that visual attention was predominantly focused on the central and top areas of the artwork, which garnered greater interest, suggesting that participants were more engaged with these locations. Areas characterized by complexity or significant visual prominence, such as the image's center and the vividly colored sections, garnered increased attention. The focused gaze spots indicated participants' meticulous examination of certain regions, whereas the more dispersed distribution signified fast scanning activity. The heat map analysis results indicate certain regions of concentrated visual attention when participants see AI-generated artworks, often characterized by great complexity and visual impact. This data facilitates a deeper comprehension of customer impressions and assessments of AI-generated artwork.

Figure 3(b) illustrates the allocation of participants' gaze duration across various parts of the artwork. A more vibrant hue signifies an extended gaze duration, indicating that the area is more visually attractive to the participants. Figure 3(b) indicates that areas with extended gaze durations were predominantly located in the central and upper sections of the artworks, which typically had more intricate visual components or pronounced color contrasts that captivated participants' attention for an extended duration. The central portion had the most brightness, indicating that participants allocated the most time there, maybe due to the artwork's design characteristics, including symmetry, color contrast, and content richness. The deeper hues in some peripheral regions suggested that these areas exhibited reduced attention spans, with individuals likely engaging in superficial scanning rather than thorough examination.

### Analysis of eye movement data results

The article found crucial visual behavior patterns by examining participants' eye movement data. Participants were required to observe each artwork for the entire 30 seconds. There was no option to skip to the next artwork. Occasional gaps in gaze data reflect brief blinks or slight distractions, which were accounted for during data preprocessing by filtering out non-informative intervals. Participants concentrated predominantly on the central and top sections of the artwork. These regions had more intricate and engaging visual features that garnered greater attention from the participants. The distribution of gaze time revealed that the majority of participants focused their attention predominantly on the middle and upper ranges, suggesting a heightened interest in these areas and prolonged observation. Visual path analysis: Participants' visual trajectories exhibited distinct stage features, often commencing from the bottom left corner and then advancing to the top right corner. This discovery indicates a certain pattern of visual movement when people see artworks.

### Influence of visual behavior on perception

A comparative investigation of several visual behavior patterns was done to enhance understanding of their influence on perception. The article examines the impact of visual behavior on perception, concentrating on the following aspects: Participants exhibiting a greater number of gaze points typically demonstrated a more profound observation and comprehension of the artwork, along with an elevated perceptual assessment. Participants exhibiting extended gaze length had a heightened awareness for the artwork's features, resulting in elevated perceptual scores. Participants exhibiting more intricate and diverse sight pathways often possessed a more holistic understanding of the artwork, resulting in better perception ratings.

**Figure 4.**
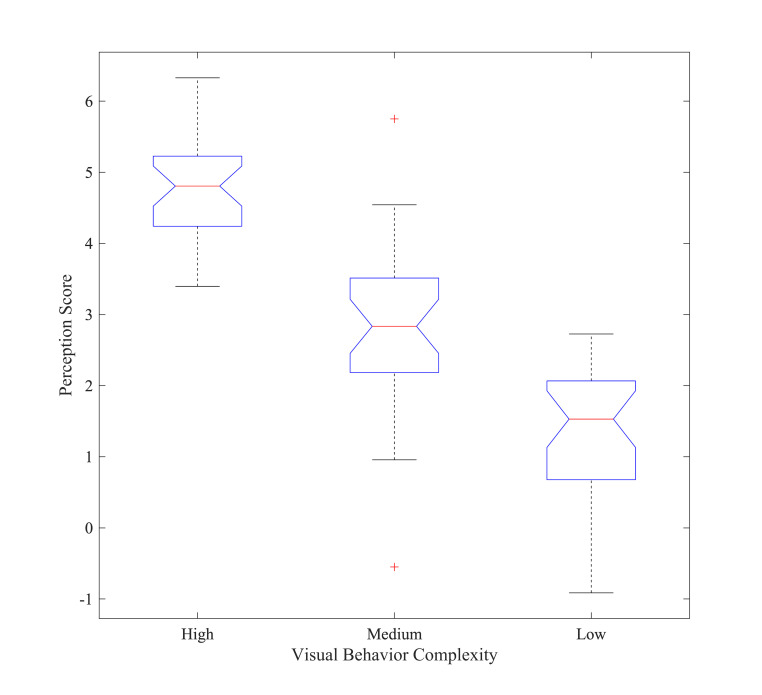
Comparison of the effects of different visual behavior patterns on perception

The classification of visual trajectory complexity (Figure 4) was based on the number of gaze points, the length of saccades, and the diversity of visual paths. High complexity reflects more gaze points and diverse saccades across different regions, while low complexity indicates fewer gaze points with shorter saccades. This classification aligns with the methodology described in Section.

Figure 4 depicts a comparison of the impacts of various visual behavior patterns on perception. Visual activity patterns of high complexity were associated with the highest perceptual evaluations, followed by medium complexity, while low complexity received the lowest ratings. The findings indicate that participants' intricate and varied visual activity patterns influenced their perceptual evaluations of the AI-generated artwork, suggesting that complex visual trajectories and prolonged gaze durations may augment participants' engagement with and comprehension of the artwork. The image illustrates that the perceptual assessments of participants exhibiting high-complexity visual activities varied between 4 and 6. Participants exhibiting a greater number of gaze locations, extended gaze length, and intricate viewing trajectories had the highest perceptions and evaluations of the AI-generated artwork. Intricate and diverse visual behavior patterns markedly enhanced participants' engagement and comprehension, therefore elevating their perceptual evaluations of the artwork. Participants exhibiting medium-complexity visual actions received perceptual scores between 2 and 4. Participants exhibited moderate gaze points and durations, along with reasonably uncomplicated visual trajectories. Participant perceptual evaluations for low-complexity visual behavior varied from 0 to 2, with an average value of roughly 1. Individuals exhibiting the low-complexity visual behavior pattern had a reduced number of gaze locations, abbreviated gaze time, and uncomplicated visual trajectories. Participants in this group exhibited less comprehension and interest in AI-generated items, resulting in lower perceptual ratings.

### Results of BERT-based sentiment analysis

The paper used the BERT model to examine participants' emotional reactions following their observation of AI-generated artwork, focusing primarily on emotional categorization outcomes and the examination of emotional intensity. These investigations offer a comprehensive knowledge of customers' emotional reactions to AI-generated artwork and their intensity, supplying empirical proof for consumer perception and approval.

### Mood Color Analysis

The emotion categorization process can be represented as follows:

**(20):**
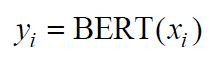


where $x_{i}$ denotes the first i text input and $y_{i}$ denotes the corresponding sentiment category output.

The calculation of emotional intensity can be expressed as:

**(21):**
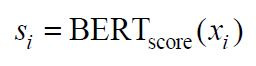


where $s_{i}$ denotes the sentiment intensity score of the first i text.

**Figure 5.**
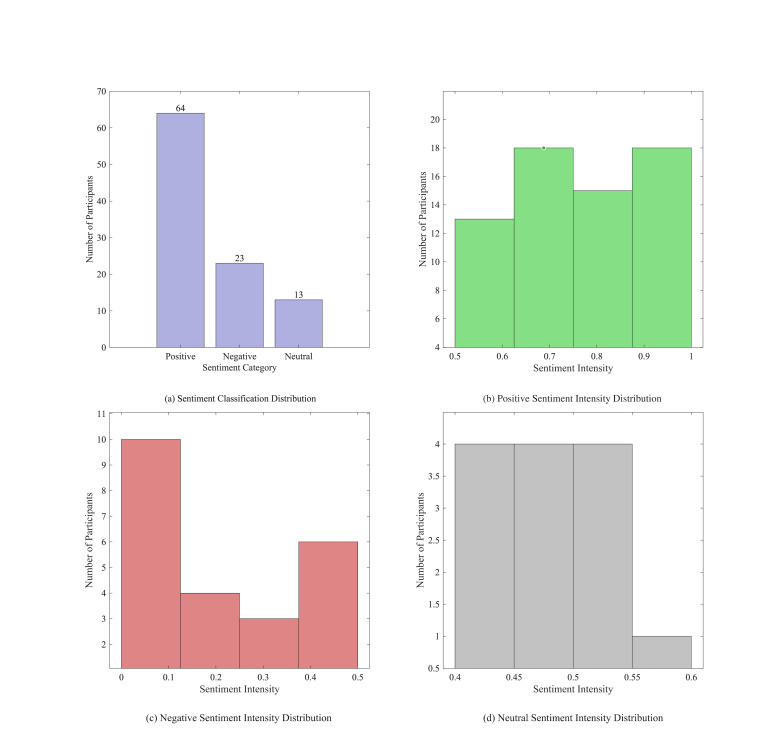
Emotional categorization and corresponding intensity distribution

The gathered emotional data were initially classified, with the emotional reactions segmented into three categories: positive, negative, and neutral. The article also examined the strength of emotional reactions based on emotion categorization. The emotional intensity was designated as the emotion score, and the intensity of each emotional reaction was computed using the BERT model. The results of emotion categorization illustrate the distribution of various emotion categories and the strength of emotions within each category, as seen in Figure 5.

Figure 5(a) displays a bar chart illustrating the categorization of emotions, with 64% positive, 23% negative, and 13% neutral sentiments toward the artwork, respectively. This outcome signifies that the participants' emotional reactions predominantly centered on pleasant sentiments. The AI-generated artwork successfully elicited favorable emotional responses in the majority of instances, signifying a substantial degree of acceptance. Secondly, Figure 5(b) illustrates the intensity distribution of positive emotion categories, with intensity values predominantly ranging from 0.5 to 1.0. The picture illustrates that the strength of the majority of positive emotional reactions is predominantly in the upper range, signifying that participants exhibited robust positive affective responses to the AI-generated artwork. This discovery further substantiates that AI-generated artwork effectively evokes robust favorable emotional reactions. Figure 5(c) illustrates the distribution of negative affect intensity, with values concentrated between 0.0 and 0.5. The findings indicate that the magnitude of negative emotional reactions is minimal, with the majority of such responses occurring between 0.2 and 0.4. This suggests that while there are some negative emotional responses, their strength is comparatively mild, indicating that the adverse emotional impact of the AI-generated artwork is minimal. Figure 5(d) depicts the intensity distribution of neutral emotions. The intensity levels are centered within the range of 0.4 to 0.6. The figure indicates that the strength of the neutral emotional reaction is predominantly situated in the mid-range of values, signifying that individuals exhibit a balanced neutral affective response to the AI-generated artwork, devoid of pronounced positive or negative inclinations. In conclusion, the examination of emotional categorization and intensity reveals that participants exhibit predominantly positive emotional reactions to AI-generated artwork, with a heightened intensity of these good emotions. Negative emotional reactions were less frequent and of diminished intensity, whereas neutral affective responses were equilibrated. The results demonstrate that AI-generated artwork have a significant capacity to evoke good emotions and efficiently capture participants' interest and attention.

### Aesthetic judgments in texts

**Figure 6.**
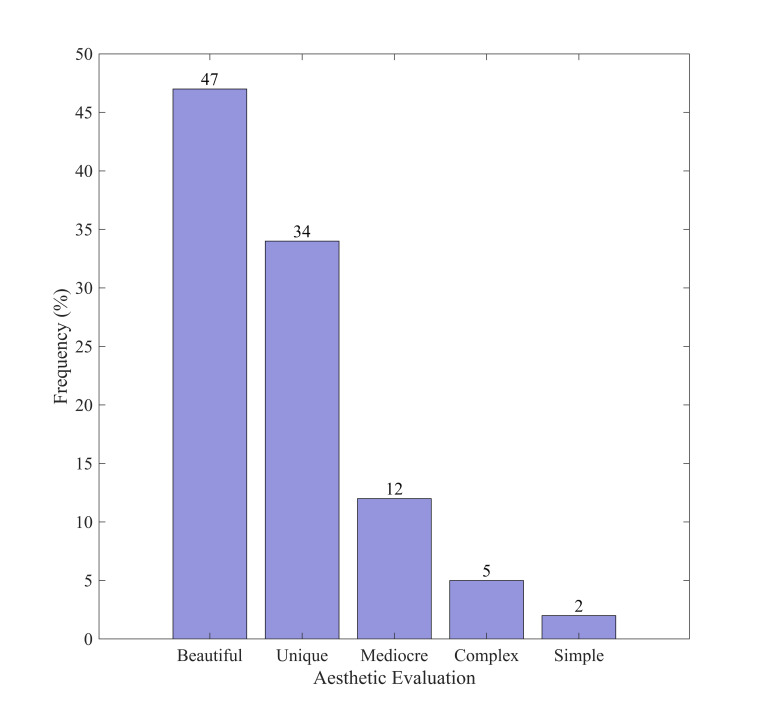
Frequency distribution of different aesthetic evaluations

Extracting and evaluating aesthetic assessments of participant textual data using the BERT model. Sentiment analysis of extensive internet reviews is employed to comprehend customers' aesthetic assessments of AI-generated artworks, therefore offering empirical assistance for investigating the acceptability and aesthetic merit of AI art. The pre-processed textual data undergoes feature extraction via a pre-trained BERT model to produce a profound semantic representation of each text. The text data are classified into several aesthetic rating categories using sentiment classifiers based on feature extraction. The article chooses "beautiful," "mediocre," "peculiar," "complex," "simple," and "beautiful." Five aesthetic judgment categories—"beautiful," "mediocre," "peculiar," "complex," and "simple"—were chosen for investigation. Subsequently, the intensity score for each aesthetic assessment was computed using the BERT model to measure the intensity of each judgment. Following the extraction of aesthetic evaluations, the frequencies of various categories are evaluated to comprehend customers' general aesthetic inclination towards AI-generated artworks. Figure 6 depicts the frequency distribution of several aesthetic assessments.

Figure 6 indicates that "beautiful" ratings are the most frequent, comprising 47% of the total. Subsequently, "peculiar" and "mediocre" represent approximately 34% and 12%, respectively. Evaluations of aesthetics categorized as "complex" and "simple" occurred with less frequency. The findings indicate that the majority of participants assessed the AI-generated artwork as possessing significant aesthetic value, recognizing it as both beautiful and unique. The high frequency of the term "peculiar" suggests that participants acknowledged the AI artworks for their creativity and novelty.

### Examination of Emotional Outcomes

Emotional classification results indicate that participants predominantly exhibited positive emotional responses, representing the largest percentage. The elevated proportion of positive emotions suggests that the AI-generated artwork successfully elicits positive emotional responses from participants. The distribution of emotional intensity among participants predominantly fell within the medium to high range, indicating that their emotional responses to the AI-generated artwork were not only largely positive but also characterized by significant intensity. The analysis of emotional intensity demonstrates that AI-generated artwork possesses significant emotional triggering capabilities. The frequency distribution of aesthetic evaluation revealed that participants predominantly categorized the AI-generated artworks as "beautiful" and "peculiar," suggesting a strong recognition of their visual appeal and innovative qualities.

### Connection between emotional response and aesthetic assessment

**Figure 7.**
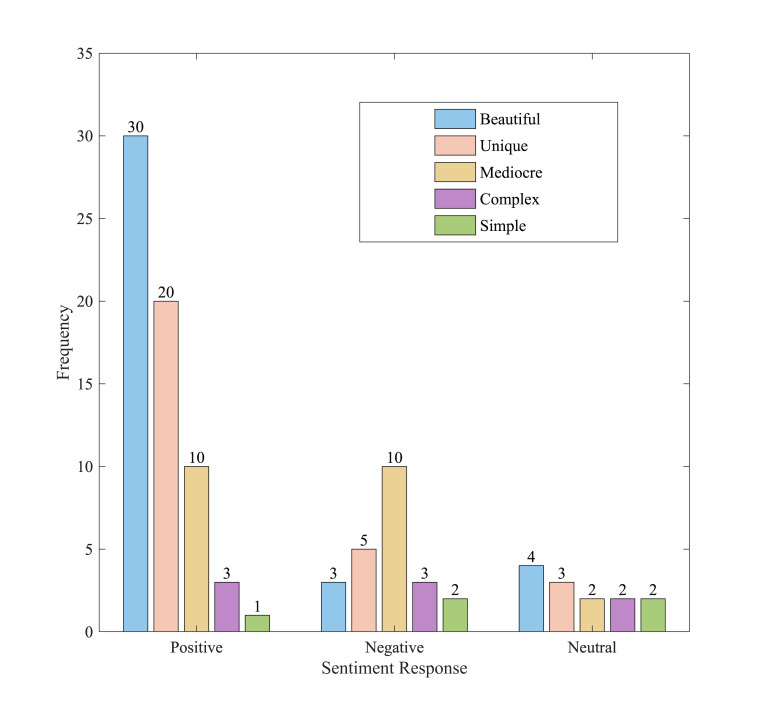
Distributional relationship between emotional response and aesthetic evaluation

The article presents a comparative analysis of aesthetic evaluations in relation to varying emotional responses, aiming to enhance the understanding of their interrelationship. Figure 7 illustrates the frequency of participants' aesthetic evaluations across various emotional states. Participants' aesthetic evaluations of the AI-generated artworks, under positive affective responses, predominantly fell within the categories of "beautiful" and "peculiar". The findings indicate that positive affective responses can markedly enhance participants' aesthetic evaluations of artworks, particularly regarding beauty and strangeness. Participants' aesthetic evaluations under negative affective responses predominantly centered on the categories of "mediocre" and "peculiar". Negative affective responses diminished participants' aesthetic evaluations of artworks, particularly in the mediocre and complex categories. Participants' aesthetic evaluations were more balanced when experiencing neutral affective responses. This finding indicates that neutral affective reactions exert a diminished influence on aesthetic evaluations, resulting in relatively uniform assessments across categories.

Summarizing Figure 7, positive affective responses notably elevated participants' assessments of the beauty and strangeness of the AI-generated artwork, suggesting that such responses improve recognition and enjoyment of the artwork. Negative affective responses primarily centered on the assessment of mediocrity and complexity, indicating that such responses diminish participants' overall evaluation and acceptance of the artwork. Neutral affective responses exerted minimal influence on aesthetic evaluations, resulting in more balanced assessments across categories. This suggests that neutral affective responses do not substantially alter participants' aesthetic judgments.

### Consumer acceptance

**Figure 8.**
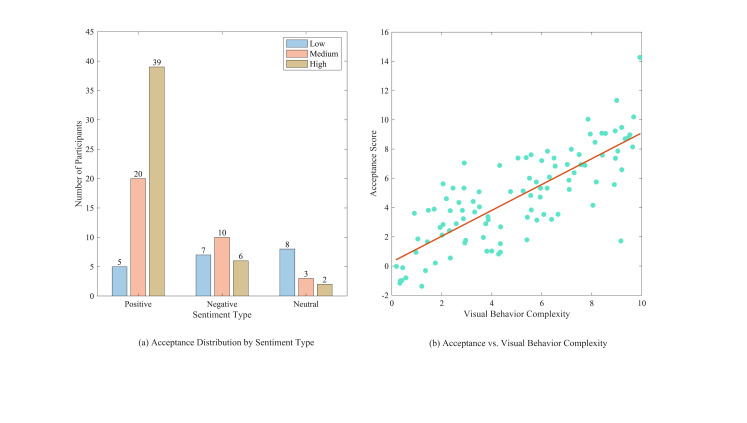
Relationship of acceptance with emotional type and visual behavior

The analysis of participants' eye-tracking data revealed a significant correlation between the complexity of visual behaviors and consumer acceptance. Complex and varied visual behavior patterns are typically associated with increased acceptance of artwork. The article quantitatively analyzed gaze points, gaze duration, and sight paths of visual behaviors, comparing these metrics with consumer acceptance, as illustrated in Figure 8.

Figure 8(a) indicates that participants' acceptance significantly increased in the presence of positive affective responses. Positive affective responses enhance consumers' recognition and enjoyment of AI-generated artwork. Participants demonstrated significantly lower acceptance when they displayed negative affective responses. Negative affective responses diminish consumer acceptance of AI-generated artwork. The acceptance of participants remained stable, showing no significant increase or decrease when neutral affective responses were displayed. This indicates that neutral affective responses exert a diminished influence on consumer acceptance. Figure 8(a) illustrates that a majority of participants exhibited high acceptance, suggesting that the AI-generated artwork achieved overall consumer acceptance. Figure 8(b) illustrates the correlation between consumer acceptance and visual behavior. The scatterplot and regression analysis indicate a positive correlation between complex visual behavior patterns and high acceptance. The complexity of visual behavior correlates positively with the consumer acceptance score of AI-generated artwork. The results indicate that complex visual behaviors significantly enhance consumers' interest in and acceptance of AI-generated artwork. Positive affective responses can enhance consumer acceptance, whereas negative affective responses diminish it, and neutral affective responses exert a lesser influence.

### Analysis of perceived artistic quality

**Figure 9.**
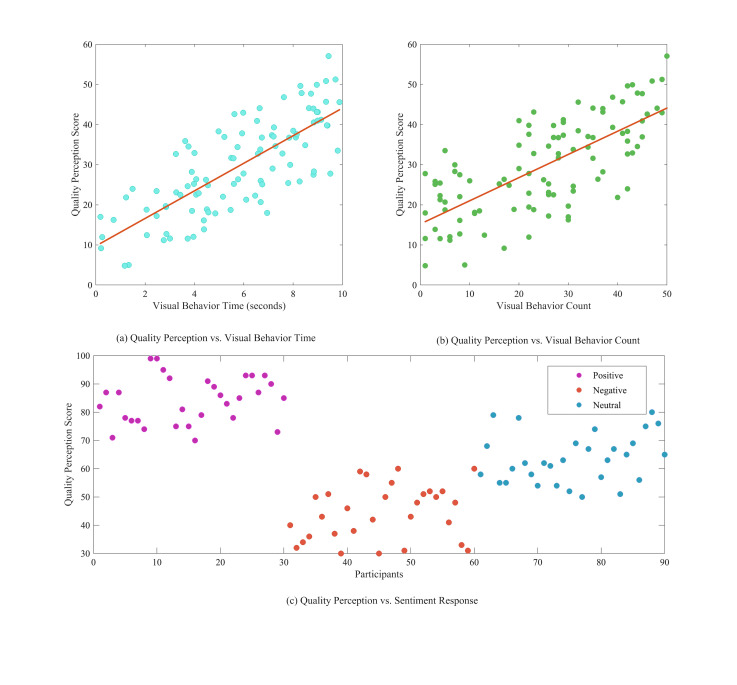
Outcome effects of visual behavior and emotional responses on perceived artistic quality

A. The impact of visual behavior on the assessment of artistic quality

Visual behavior significantly affects the perception of artwork. Analysis of participants' eye-tracking data revealed that gaze duration and the number of gazes significantly influenced artwork perception. Longer gaze duration and a greater number of gazes are typically associated with an elevated perception of art quality. Figure 9(a) illustrates the correlation between visual behavior and the perception of art quality, highlighting the association between the duration of visual behavior and the assessment of art quality. Scatterplot and regression analyses indicate a significant positive correlation between gaze duration and the perception of art quality; specifically, increased gaze duration corresponds to higher ratings of perceived artwork quality by participants. Figure 9(b) illustrates the correlation between the frequency of visual behaviors and the perception of art quality. The scatterplot and regression analysis indicate a significant positive correlation between the number of gazes and perceived artistic quality; as the number of gazes increases, so does the participant's rating of the artwork's perceived quality.

B. The influence of emotional responses on the assessment of artistic quality

Emotional responses significantly influence the perception of artistic quality. The analysis of participants' affective responses revealed that positive affective responses correlated with elevated perceptions of art quality, whereas negative affective responses were associated with diminished perceptions of art quality. Figure 9(c) indicates that participants' perception of artistic quality was significantly elevated in the presence of positive affective responses. This indicates that positive emotional responses improve participants' ability to identify and assess the quality of the artwork. Participants' perceptions of art quality significantly diminished in the presence of negative affective responses. This indicates that negative affective responses diminish participants' ability to identify quality in the artwork. Furthermore, participants' perceptions of art quality remained stable, showing no significant increase or decrease when they displayed neutral affective responses. This indicates that neutral affective responses exert a diminished influence on perceptions of art quality.

## Discussion

This article examines consumer perception and acceptance of AI-generated artwork utilizing eye-tracking techniques and a BERT-based sentiment analysis approach, highlighting the influence of visual behavior and emotional responses on the perceived quality of the art.

### Main Findings of the Study

This study revealed several significant findings regarding the relationship between visual behavior, emotional responses, and consumer acceptance of AI-generated art. The results showed that longer fixation durations and complex gaze paths were associated with higher levels of visual engagement, confirming prior research that links visual attention to consumer interest (5). In addition, our sentiment analysis identified nuanced emotional responses to different artistic elements, with positive sentiment aligning with vibrant colors and symmetry, supporting findings from prior studies on visual aesthetics (1). By integrating eye-tracking data with sentiment analysis, our study provides a more holistic view of consumer perception, extending previous work that primarily focused on either visual behavior or emotional response in isolation. These findings contribute to the theoretical framework of AI art perception by offering empirical evidence on how visual and emotional factors jointly influence acceptance. Moreover, the innovative use of BERT models fine-tuned with domain-specific data offers new methodological insights for future research on sentiment analysis in digital art evaluation.

### Practical and theoretical consequences

This study uses eye-tracking and BERT-based sentiment analysis to examine how visual activity and emotional reactions affect art quality and customer approval. AI art theoretical research gains fresh views and methodologies from this work. This study examines how complex visual behaviors and emotional reactions impact customers' aesthetic evaluations and acceptance of artwork. Complex and diverse visual behaviors and positive emotional reactions can considerably boost customer approval of AI-generated artwork, an article found. Evidence for AI art production and marketing comes from this finding. To improve artwork appeal and marketability, artists should optimize visual components and evoke pleasant feelings. This discovery may also be used to create individualized artwork advertising plans for distinct emotional reactions to boost AI art market penetration. Artists and promoters may enhance user experience by understanding how visual behavior and emotional reactions affect art quality. Improve user happiness and identification with AI-generated artworks by developing more appealing visual components and boosting positive emotions. This practical use boosts AI artworks' market viability and encourages AI technology in art production.

### Research limits and future directions

Despite this study's crucial findings on public views and acceptance of AI-generated artwork, future research must overcome and expand its limitations.

Study sample size and representativeness: The small sample size and concentration on specific groups may restrict generalizability of findings. To increase representativeness and external validity, future research should include people of varied ages, genders, cultures, and education levels. Experiment environment constraints: Despite assuring data quality, this study's studies were mostly done in controlled contexts and may not fully reflect participants' real-life behaviors and sentiments. To boost ecological validity, future study might use virtual reality or natural surroundings to imitate real-life circumstances.

Future study might build a multimodal sentiment analysis model including eye tracking, facial expressions, voice analysis, and physiological inputs. This will assist better capture consumers' emotional responses, revealing how emotions affect creative quality assessment and acceptance. However, future study can measure customers' behavioral and emotional responses to AI-generated artworks over time. This will assist define customers' emotions and behavior across time and situations and enable AI art production and marketing strategy improvement.

### Conclusion

Eye-tracking and BERT-based sentiment analysis are used to examine consumer perception and acceptance of AI-generated artworks, demonstrating how visual behavior and emotional reactions affect art quality. The article's major findings: Visual behavior complexity positively correlates with artistic quality. Eye-tracking study demonstrated that visual behavioral variables like gaze spots, gaze duration, and sweep route length greatly impact artistic quality. Emotional response significantly affected art quality perception. Through BERT-based sentiment analysis, positive affective responses greatly boosted participants' quality evaluation of AI-generated artwork, whereas negative affective responses dramatically diminished it. Art quality judgment was less affected by neutral emotional reactions. The participants' visual variety and positive emotional responses increased their approval of the AI-generated artwork. Combining the improved methods of deep Gaussian process and dynamic Bayesian optimization improved the accuracy of sentiment analysis and the robustness of the model, proving that the optimization strategy improved model performance and practicality.

This study uses eye tracking and BERT-based sentiment analysis to show how visual activity and emotional reactions affect AI-generated artwork perception and acceptance. The findings enhance AI art comprehension and give a scientific foundation and practical recommendations for future development and promotion.

This study has shed light on customers' view and acceptance of AI-generated artworks, however many areas need additional study. To disclose the multifaceted elements impacting consumer perception and acceptance and encourage AI art, future study should deepen and extend sample size and diversity and multimodal sentiment analysis.

### Ethics and Conflict of Interest

The author(s) declare(s) that the contents of the article are in agreement with the ethics described in http://biblio.unibe.ch/portale/elibrary/BOP/jemr/ethics.html and that there is no conflict of interest regarding the publication of this paper.

### Acknowledgements

The authors thank all the participants in this study for their time and willingness to share their experiences and feelings.
